# Ibuprofen in Therapeutic Concentrations Affects the Secretion of Human Bone Marrow Mesenchymal Stromal Cells, but Not Their Proliferative and Migratory Capacity

**DOI:** 10.3390/biom12020287

**Published:** 2022-02-10

**Authors:** Agnieszka Kulesza, Katarzyna Zielniok, Jakub Hawryluk, Leszek Paczek, Anna Burdzinska

**Affiliations:** 1 Department of Immunology, Transplantology and Internal Diseases, Faculty of Medicine, Medical University of Warsaw, Nowogrodzka 59, 02-006 Warsaw, Poland; akulesza@wum.edu.pl (A.K.); jakub.m.hawryluk@gmail.com (J.H.); leszek.paczek@wum.edu.pl (L.P.); 2 Department of Clinical Immunology, Faculty of Medicine, Medical University of Warsaw, Nowogrodzka 59, 02-006 Warsaw, Poland; katarzyna.zielniok@wum.edu.pl; 3 Department of Bioinformatics, Institute of Biochemistry and Biophysics, Polish Academy of Sciences, Pawińskiego 5A, 02-106 Warsaw, Poland

**Keywords:** ibuprofen, human mesenchymal stromal cells, bone marrow, secretion, prostaglandin E2, cyclooxygenase, immunomodulation, regeneration

## Abstract

Mesenchymal stromal cells (MSCs) are able to modulate the immune system activity and the regeneration processes mainly through the secretion of multiple soluble factors, including prostaglandin E2 (PGE_2_). PGE_2_ is produced as a result of cyclooxygenases (COX) activity. In the present study, we investigated how ibuprofen, a nonselective COX inhibitor, affects the proliferation, migration and secretion of human bone marrow MSCs (hBM-MSCs). For this purpose, six hBM-MSCs populations were treated with ibuprofen at doses which do not differ from maximum serum concentrations during standard pharmacotherapy. Ibuprofen treatment (25 or 50 µg/mL) substantially reduced the secretion of PGE_2_ in all tested populations. Following ibuprofen administration, MSCs were subjected to proliferation (BrdU), transwell migration, and scratch assays, while its effect on MSCs secretome was evaluated by Proteome Profiler and Luminex immunoassays. Ibuprofen did not cause statistically significant changes in the proliferation rate and migration ability of MSCs (*p* > 0.05). However, ibuprofen (25 µg/mL for 3 days) significantly decreased mean secretion of: CCL2 (by 44%), HGF (by 31%), IL-6 (by 22%), VEGF (by 20%) and IL-4 (by 8%) compared to secretion of control MSCs (*p* < 0.05). Our results indicate that ibuprofen at therapeutic concentrations may impair the pro-regenerative properties of hBM-MSCs.

## 1. Introduction

Mesenchymal stromal cells (MSCs) are currently one of the most extensively studied mammalian cell populations [1]. The easy availability, high proliferation potential and low immunogenicity are features that make MSCs an ideal tool for regenerative medicine. From a clinical point of view, an essential property of MSCs is also an ability of migration to the site of injury, allowing them to act locally in the inflamed area [2]. Therefore, numerous studies are being conducted on the use of MSCs in the treatment of various diseases, such as myocardial infarction, damage of central nervous system or peripheral nerves, diabetes, liver cirrhosis, skeletal muscle injures, autoimmune diseases, graft versus host disease and many others [3,4]. However, the detailed mechanisms of the therapeutic action of MSCs are not fully understood. Initially, it was considered that their ability to differentiate into cells of various tissues seemed to be the most promising [5,6,7]. Nowadays, it is postulated that the main regenerative potential of MSCs is related to the stimulation of endogenous regenerative mechanisms and immunomodulatory activity [8]. MSCs can dynamically respond to the microenvironment of the injury by altering their secretory profile to restore homeostasis. These cells are believed to act as specific coordinators of tissue repair by interacting with immune cells which were reviewed by Andreeva et al. [9].

In response to ongoing inflammation, MSCs release various soluble trophic factors, such as cytokines, chemokines, hormones and growth factors that affect tissue repair processes [10]. These molecules exhibit various properties: anti-apoptotic and pro-angiogenic e.g., vascular endothelial growth factor (VEGF), hepatocyte growth factor (HGF), basic fibroblasts growth factor (bFGF) [11], immunomodulatory e.g., interleukin (IL)-6, IL-10, transforming growth factor β (TGF-β), prostaglandin E_2_ (PGE_2_), indoleamine 2, 3-dioxygenase (IDO) [12], chemotactic e.g., CC chemokine ligand (CCL)-2 (monocyte chemoattractant protein (MCP)-1), CXC chemokine ligand (CXCL)-8 (IL-8), CXCL-12 (Stromal-Derived Factor (SDF)-1) [13] and others [14]. Among the factors secreted by MSCs, PGE_2_ seems to be of particular importance. PGE_2_ is one of the most important lipid mediators produced from arachidonic acid by the enzyme prostaglandin cyclooxygenase (COX). Research on COX inhibitors has confirmed that COX-2, one of the COX isoforms, is required for PGE_2_ production [15]. MSCs constitutively express both COX-2 and PGE_2_, and their expression levels increase significantly in an inflammatory environment [16]. PGE_2_ is considered one of the essential elements in forming anti-inflammatory response of MSCs through pleiotropic effects on many immune cells, like regulatory T cells, dendritic cells, and Natural Killer cells [16,17,18]. Moreover, the role of PGE_2_ in the polarization of macrophages to the anti-inflammatory M2 phenotype has been described in the literature [19]. It is also believed that PGE_2_ may play an important role in regulating MSCs proliferation. It has been suggested that PGE_2_ may affect the proliferative activity of the MSCs by acting on the COX-2 pathway with chemical inhibitors or siRNA. Suppression of PGE_2_ production with COX-2 inhibitors or inhibitors of mPGES-1 (an enzyme for PGE_2_ synthesis) resulted in a consistent reduction in proliferation of human MSCs [20]. The role of PGE_2_ in MSCs migration has also been indicated. PGE_2_ has been shown to stimulate the migration of various cell types through the prostaglandin E_2_ receptor (EP_2_) [21]. It is considered that PGE_2_/EP_2_ is crucial for MSCs homing to injured tissue after transplantation, which may be important for improving the efficacy of cell therapies [22]. It is also suggested that the level of PGE_2_ secretion by MSCs correlates with their immunomodulatory properties. An association between the ability of MSCs to inhibit the production of either tumor necrosis factor (TNF)-α or interferon (IFN)-γ and COX-2 expression has been demonstrated [23]. Altogether, these data indicate that PGE_2_ is an important factor affecting many processes involved in the MSCs immunomodulation.

One of the most commonly used drugs worldwide are non-steroidal anti-inflammatory drugs (NSAIDs), which analgesic and anti-inflammatory properties based on the inhibition of two isoforms of cyclooxygenase, COX-1 and COX-2, thereby reduce the synthesis of prostaglandin, including PGE_2_ [24]. Previously, our group has demonstrated that drugs in therapeutic doses can affect the MSCs behavior [25]. Due to the critical role of PGE_2_ in the immunomodulatory properties of MSCs, there is a risk that inhibiting its production by NSAIDs may adversely affect the body’s natural defense mechanisms. There are reports in the literature of negative effects of NSAIDs on the recovery of various tissues, including bone [26,27], muscle [28,29] and tendon [30,31]. The properties of NSAIDs and their effect on regenerative mechanisms also play a significant role in cell therapy. These drugs are used to relieve pain and reduce inflammation in cell therapy procedures [32]. Some studies suggest that NSAIDs may adversely affect the viability and regenerative potential of transplanted cells [33,34,35]. Therefore, we decided to investigate whether ibuprofen, the most popular drug of the NSAIDs class, could affect the properties of MSCs, especially by altering their proliferation, migration and secretion.

## 2. Materials and Methods

### 2.1. Cell Isolation and Culture

Human Bone Marrow-Derived Mesenchymal Stromal Cells (hBM-MSCs) were isolated from bone marrow (BM) aspirates obtained during standard orthopaedic surgeries with written consent from each patient. Donors were both sexes, in the age 18–68, without chronic diseases. The procedure was approved by the Local Bioethics Committee (number KB/115/2016). Cells were isolated as we previously described [36]. Briefly, shredded bone marrow samples (approximate volume 2–8 mL) were diluted 4 times in Phosphate-Buffered Saline (PBS; Invitrogen, Thermo Fisher Scientific, Waltham, MA, USA), centrifuged and seeded on one (if less than 4 mL of BM) or two (if more than 4 mL BM) Ø100 mm plastic culture dish (BD Primaria™; Corning^®^, Glendale, CA, USA) in standard growth medium (GM) consisting of low glucose Dulbecco’s modified Eagle’s medium (DMEM; Biowest, Riverside, MO, USA) completed with 10% Foetal Bovine Serum (FBS; Biowest, Riverside, MO, USA) and 1.5% (*v/v*) antibiotic–antimycotic solution (penicillin-streptomycin-amphotericin B; Invitrogen, Thermo Fisher Scientific, Waltham, MA, USA). Cell culture was performed under standard conditions: 37 °C, 5% CO_2_, 95% humidity. After 4 days of culture, the GM was replaced and first fibroblast-like, adherent cells were observed. In further culture, medium was changed every other day, until sub-confluence was achieved. Then, cells were passaged by harvesting 0.25% trypsin-0.02% EDTA solution (Sigma-Aldrich, Saint Louis, MO, USA). In the growth phase, the cells were seeded in a density of 4 × 10^3^/cm^2^. After the 2nd or the 3rd passage, the cells were cryopreserved for further analyses. Due to occurrence of in vitro cellular aging described in literature [37], all experiments were performed between 4–6 passages.

### 2.2. hBM-MSCs Identification

To confirm phenotype of isolated cells, every hBM-MSCs population at the 3rd passage was identified by flow cytometry and multilineage differentiation capacity according to the phenotypic signature described by The Mesenchymal and Tissue Stem Cell Committee of the International Society for Cellular Therapy (ISCT). The protocol of the procedure was described in detail previously by our group [38].

#### 2.2.1. Antigen Characterisation

Phenotyping of BM-MSCs by flow cytometry was conducted using BD human Mesenchymal Stem Cell Analysis Kit (BD Biosciences, San Jose, CA, USA) for evaluation the presence of CD73, CD90 and CD105 and the absence of CD11b, CD19, CD34, CD45 and HLA-DR surface antigens. Analysis was performed on BD FACS Canto II cytometer, with BD FACS Diva Software (BD Biosciences, San Jose, CA, USA).

#### 2.2.2. Multilinear Differentiation

The multipotency of isolated hBM-MSCs was demonstrated by differentiation toward 3 lines: adipogenic, osteogenic and chondrogenic. For this purpose, cells were cultured in proper differentiation medium for 3 weeks. Following this, cells were fixed with 4% paraformaldehyde and results were verified. Adipogenic differentiation was assessed by visualizing lipid droplet formation using Oil Red O staining (Sigma-Aldrich, Saint Louis, MO, USA). Osteogenic potential was confirmed by the staining of calcium deposits with Alizarin Red (Sigma-Aldrich, Saint Louis, MO, USA) solution. Chondrogenesis of MSCs was performed in 3-dimensional culture, to obtain spheroid cell pellets. The structures were then subjected to standard histological preparation and Safranin O staining.

### 2.3. Ibuprofen Treatment

For all experiments presented in this study, an ibuprofen stock at a concentration of 20 mg/mL in ethanol was used. Ibuprofen was purchased from Sigma Aldrich (Saint Louis, MO, USA). The stock solution was prepared freshly before each experimental set-up and was stored in 4 °C for a maximum of one week. According to the results of MTT assay (preliminary data, described below) and literature data, the selected concentrations of ibuprofen were 25 µg/mL (IBU25) and 50 µg/mL (IBU50) and do not differ from the maximum serum concentrations of ibuprofen during standard pharmacotherapy [39]. The duration of ibuprofen treatment for main experiments was set at 72 h. The control groups (CTRL) in all experiments were hBM-MSCs treated with an equal amount of solvent (ethanol) that was in the tested samples. The final concentration of solvent in samples achieved, respectively, 0.12% (*v/v*) in samples treated with 25 µg/mL of ibuprofen and 0.25% (*v/v*) in samples treated with 50 µg/mL of ibuprofen. Presented ethanol concentrations did not caused a cytotoxic effect, as was determined by the MTT test.

#### 2.3.1. MTT Assay

The effect of ibuprofen on hBM-MSCs viability was evaluated. For this purpose, the tetrazolium salt (MTT) reduction test was carried out. In the preliminary study, cells from 3 donors were seeded in 96-well plates at a concentration of 8 × 10^3^ cells per well. Cells were treated with different concentrations of ibuprofen (25 µg/mL, 50 µg/mL, 100 µg/mL, 200 µg/mL, 400 µg/mL, and 800 µg/mL) for respectively 24 h, 48 h, 72 h, 96 h or 120 h. Two hours before incubation ended, 5 mg/mL of MTT solution (in PBS) was added to each well (final concentration of MTT—1 mg/mL). Following this, medium was removed and 100 µL of dimethyl sulfoxide (DMSO; Sigma Aldrich, Saint Louis, MO, USA) was added. The absorbance was measured with a microplate reader (PowerWave XS, BioTek, Santa Clara, CA, USA) at a wavelength of 570 nm. The experiment was performed in triplicates. According to the obtained data, hBM-MSCs survival curves were plotted. The experiment was repeated for selected doses of ibuprofen (25 µg/mL and 50 µg/mL) on 6 populations. This time, 4 different time points were tested: 1 day (24 h), 3 days (72 h), 7 days and 10 days.

#### 2.3.2. PGE_2_ ELISA Immunoassay

To verify the effect of ibuprofen treatment at selected doses on hBM-MSCs, PGE_2_ secretion was measured with a monoclonal Prostaglandin E2 ELISA Kit (range 7.8–1000 pg/mL; Ref No. 514010, Cayman Chemical, Ann Arbor, MI, USA). For this purpose, hBM-MSCs were seeded onto 12-well plates to achieve 90% confluency the next day (6 × 10^4^ cells per well). Cells were then preconditioned with 25 ng/mL of IFN-γ (Sigma-Aldrich, St. Louis, MO, USA). After 24 h, cells were rinsed with PBS and GM was replaced with OptiMEM™ Medium (no phenol red; Gibco, Thermo Fisher Scientific, Waltham, MA, USA) containing 4% (*v/v*) FBS and 1% (*v/v*) penicillin-streptomycin. In experimental samples, ethanol (for control cells) or ibuprofen at concentrations of 25 or 50 µg/mL was added. Cells were incubated for 24 h at 37 °C and 5% CO_2_. After the incubation, supernatants were collected and centrifuged for 5 min at 4500 rpm to remove any cellular debris and then transferred to new Eppendorf tubes. Prepared samples were immediately frozen at −80 °C until the day of analysis. ELISA assay was performed according to the manufacturer’s protocol. Samples were analysed in duplicates. Cells used in this experiment were derived from 6 independent donors.

### 2.4. BrdU Proliferation Assay

For this experiment, hBM-MSCs were seeded in 96-well plates (at a density of 4 × 10^3^ cells per well) and treated with ibuprofen at concentrations of 25 or 50 µg/mL or ethanol (at adequate concentration, as control groups) for 72 h. Medium was changed every 24 h with a new dose of ethanol/ibuprofen added. The cell proliferation rate was evaluated using a colorimetric, 5-bromo-2′-deoxyuridine (BrdU) ELISA immunoassay (Roche, Indianapolis, IN, USA), which was based on the measurement of BrdU incorporation during DNA synthesis in tested cells. After 60 h of indicated treatment, experimental medium was replaced and BrdU labeling reagent (100 µM) was added to each well. Cells were further incubated for the next 16 h at 37 °C, 5% CO_2,_ and 95% humidity (BrdU pyrimidine analogue is incorporated into the DNA). Next, an assay was performed according to the manufacturer’s instructions. The reaction products were quantified by measuring absorbance at a wavelength of 370 nm with the reference wavelength at 492 nm (PowerWave XS, BioTek, Santa Clara, CA, USA). Samples were analysed in quadruplicates. The experiment was performed on the cells derived from 6 independent donors.

### 2.5. Transwell Migration Assay

This assay was designed to evaluate the impact of ibuprofen on hBM-MSCs secretion in terms of stimulating the targeted migration. For this test, each 6-cell populations (one population- cells originating from one donor) were divided into two parts upon detachment. Part of each cell population was seeded separately on 12-well plates in a density of 4 × 10^4^ cells per well and were then preconditioned with 25 ng/mL of IFN-γ (MilliporeSigma, St. Louis, MO, USA). After 24 h, cells were treated with 50 µg/mL of ibuprofen for the following 24 h or with an adequate amount of solvent (control cells). These cells became “stimulating populations”. The other part of the cells were pooled with cells from other donors and stained with red fluorochrome DilC18(5)-DS (DID, 1,1′-Dioctadecyl-3,3,3′,3′-Tetramethylindodicarbocyanine-5,5′- disulfonic acid, Ex = 650 nm, Em = 670 nm; AAT Bioquest; Sunnyvale, CA, USA). This fused population constituted a “common migrating population” which was seeded on each cell culture insert with 8 µm pore size in a density of 2 × 10^4^ cells per insert (ThinCert™, Greiner Bio-One, Kremsmünster, Austria). Cells were cultured for the next 72 h at 37 °C, 5% CO_2_, and 95% humidity. The pore size used in this experiment allowed stained cells to migrate to the bottom compartment of the well, in response to the soluble factors secreted from ibuprofen treatment or to control hBM-MSCs placed in the basal compartment. At the end of the treatment, the inserts were removed. The cells in the basal compartment were fixed with 4% paraformaldehyde (PFA) and nuclei were stained with DAPI (0.5 µg/mL solution, 4 min, RT). The number of migrated cells (DID+ cells in the basal compartment and that fell from the insert) was determined with an automated cell imaging multi-mode microplate reader Cytation™ 3 (BioTek, Santa Clara, CA, USA). The number of cells was calculated from 9 different fields of view in each well (with the same location in each well). The results are given as the % of migrated (DID+/DAPI+) cells per number of stimulating cells (DID−/DAPI+). The experiment was performed in duplicates.

### 2.6. Cell Migration Scratch Assay—Live Cell Imaging

This assay was performed to evaluate the effect of ibuprofen on the over BM-MSCs mobility. The scratch technique, imaging and analysis methods were carried out as previously described by our group [40]. Cells were seeded onto 96-well, µCLEAR, black plates (Ref No. 655090, Grainer Bio-One, Kremsmünster, Austria) at a concentration of 1 × 10^4^/100 µL/well. Such a number ensured full confluence at the beginning of the experiment. The next day, the medium with addition of IFN-γ (25 ng/mL) was added for 24 h. MSCs were then washed once and then scratched in each well using a 200 µL tip and a sterile ruler to ensure a straight “wound” path. The assay was performed in quadruplicates. The cells were then washed once more and exposed to appropriate medium (150 µL/well): basal medium (CTRL-Opti-MEM™ supplemented with 4% FCS), basal medium with ethanol (EtOH solvent control), basal medium with 50 µg/mL ibuprofen (IBU50) or basal medium with 5 ng/mL bFGF (bFGF). The addition of bFGF was used as positive control as it is a factor known to increase both the migration capacities and proliferation rate of various cell types [41]. The plates were placed in a Cytation™ 1 Cell Imaging Multi-Mode Reader (BioTek, Santa Clara, CA, USA) under temperature—(37 °C) and CO_2_—(5%) controlled conditions. The cells underwent kinetic image capturing (bright field) in a 3 h time-lapse for 24 h. For each well, the imaging area was selected manually prior to analysis in such a way that the “scratch” was in its center and covered as a straight fragment of the “wound” as possible. Images were analysed using Gene 5 3.04 software. Due to the manual execution of the scratch and possible differences in the size of the scratch between the wells, the degree of the covering of the “wound” surface with cells was analyzed in µm^2^. For each time point, the difference between the cell-covered area at that time point and the cell-covered area at time point 0 for each well was calculated. The analysis was conducted in a “plug” covering the scratch surface with a certain margin. The use of a “plug” reduced the potential effect of artifacts that could be outside the scratch area on the analysis. Altogether, MSCs from 5 donors were used for this test.

### 2.7. Cytokine Secretion Analysis

The level of various cytokine secretion by ibuprofen treatment and control of hBM-MSCs was compared. The samples were prepared similarly to the ELISA assay (described above). BM-MSCs from 6 donors were seeded on 12-well plates (5 × 10^4^ cells per well) and cultured in standard GM to achieve 80% confluency. Cells were then treated (or not in control groups) with 25 or 50 µg/mL of ibuprofen for 72 h, with medium change after every 24 h. For the last 24 h, medium was replaced with Opti-MEM™ Medium (no phenol red; Gibco, Thermo Fisher Scientific, Waltham, MA, USA) containing 4% (*v/v*) FBS and 1% (*v/v*) penicillin-streptomycin. After 24 h, supernatants were collected, centrifuged for 5 min. at 3000 rpm and transferred to new Eppendorf tubes. Samples were then aliquoted for 3 equal samples and immediately frozen at −80 °C for further analysis (Section 2.7.2 and Section 2.7.3).

#### 2.7.1. Proteome Profiler Antibody Assay

The secretion level of over 100 human cytokines in the tested samples was determined semi quantitatively with Proteome Profiler Human XL Cytokine Array (R&D Systems, Bio-Techne, Minneapolis, MN, USA). Cell supernatants were thawed on ice. To compare secretory activity in control and ibuprofen (50 ug/mL) groups, samples from 6 donors were pooled within the study groups (CTRL and IBU50). Furthermore, the assay was performed according to the manufacturer’s instructions. This membrane-based sandwich immunoassay captures the proteins which are visualized with chemiluminescence. The signal produced was detected with the ChemiDoc MP Imaging System (Bio-Rad Laboratories Inc., Hercules, CA, USA). The results were analysed using Image Lab software (Bio-Rad Laboratories Inc., Hercules, CA, USA).

#### 2.7.2. Luminex Multiplex Immunoassay

Luminex Multiplex kits (R&D Systems, Bio-Techne, Minneapolis, MN, USA) were used for the simultaneous quantification of selected human cytokines, chemokines and growth factors: IL-1β, IL-6, IL-8, IL-10, Growth/Differentiation Factor (GDF)-15, HGF, MCP-1 and VEGF in cell supernatants collected previously and frozen in −80 °C (preparation procedure describe above). The analysis was performed according to the manufacturer’s specification and analysed by the Luminex 200 Instrument (Thermo Fisher Scientific, Waltham, MA, USA). The experiment was performed in duplicates on cell populations from 6 donors.

#### 2.7.3. BCA Protein Assay

The total protein concentration in the tested samples was measured using the colorimetric BCA protein assay kit (Thermo Fisher Scientific, Waltham, MA, USA). The assay was performed in quadruplicates, with the same supernatant samples used for the Luminex analysis (originating from 6 donors). For this analysis, samples were diluted 10× with sterile ultrapure water, to obtain the final concentration of protein in the sample in the range from 20 to 2000 µg/mL (linear working range for BSA). The absorbance was measured at 562 nm on a plate reader (PowerWave XS, BioTek, Santa Clara, CA, USA). To determine the protein concentration of each sample, the standard curve was plotted.

### 2.8. Statistical Analysis

Statistical analysis was performed using STATISTICA 13.1 software (Tibco, Palo Alto, CA, USA). All values are presented as mean ± SD or mean ± SEM. To determine data distribution, the Shapiro-Wilk test was used. Statistical significance between two groups was determined by the Wilcoxon matched-pairs signed-rank test (for data with abnormal distribution) or the Student’s *t*-test (for data with normal distribution). Statistical significance between more than two groups was determined by one-way ANOVA with the post-hoc Tukey test. *p*-value < 0.05 was considered statistically significant.

## 3. Results

### 3.1. Characterization of Isolated hBM-MSCs Populations

**Figure 1:**
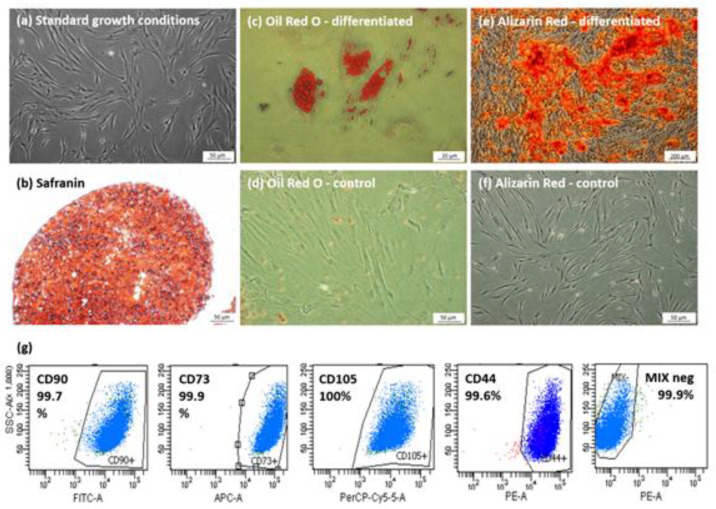
Identification of hBM-MSCs. (**a**) Untreated BM-MSCs during growth phase. Light microscopy; (**b**) chondrogenic differentiation, chondropellet stained with Safranin O; (**c**,**d**) Oil Red O staining of hBM-MSCs cultured in adipogenic (**c**) or control medium (**d**) (visible fat droplets stained in red); (**e**,**f**) Alizarin Red staining of BM-MSCs cultured in osteogenic (**e**) or control medium (**f**) (visible red calcium deposits). (**g**) Flow cytometry analysis of surface antigens on representative hBM-MSCs population. MSCs were positive for CD90, CD73, CD105 and CD44. Negative cocktail (MIX neg) consisted of CD34, CD45, CD11b, CD19 and HLA-DR; Scale bars: 50 µm (**a**–**d**); 20 µm (**c**); 200 µm (**e**).

All populations isolated from human bone marrow aspirates used in the experiments were characterized and met the criteria for human MSCs [42]. Following the Mesenchymal and Tissue Stem Cell Committee of the International Society of Cellular Therapy guidelines, cells displayed fibroblast-like morphology, adhered to the plastic and were able to form colonies (Figure 1a). To confirm multipotent properties, cells were differentiated into adipocytes, osteoblasts, and chondroblasts, and were demonstrated by adequate staining of in vitro culture (Figure 1b–f). Cytometric analysis indicated specific surface antigen expression typical for MSCs. Cells were positive for markers: CD90 (mean 98.1%), CD44 (mean 99.6%), CD73 (mean 99.9%), CD105 (mean 99.6%) and more than 98% of cells were negative for CD34, CD45, CD11b, CD19 and HLA-DR markers (Figure 1g).

### 3.2. Ibuprofen Reduces the Viability of hBM-MSCs in a Dose-Dependent but Not a Time-Dependent Manner

**Figure 2:**
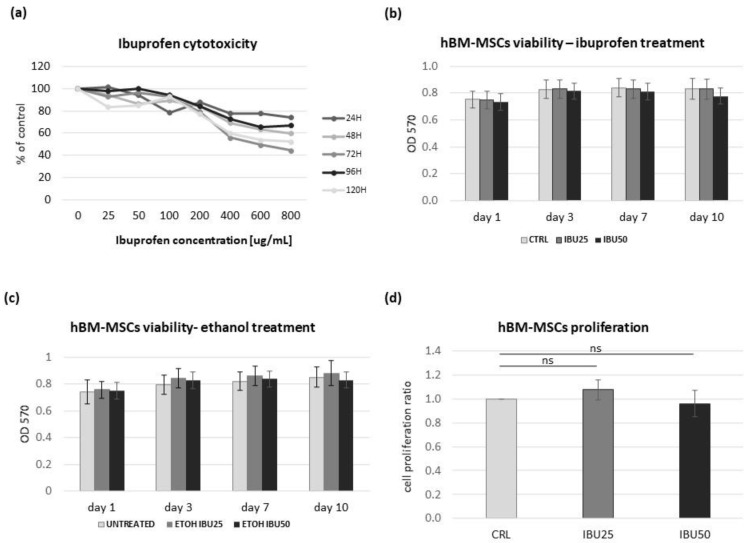
The effect of ibuprofen on the viability and proliferation of hBM-MSCs. (**a**–**c**) The effect of ibuprofen on hBM-MSCs viability was evaluated with MTT assay. (**a**) Cells from 3 donors were treated with different concentrations of ibuprofen (25 µg/mL, 50 µg/mL, 100 µg/mL, 200 µg/mL, 400 µg/mL, and 800 µg/mL) for, respectively, 24 h, 48 h, 72 h, 96 h or 120 h. The experiment was performed in triplicates. Data are presented as percentage of control cells (untreated cells analyzed after 24 h); (**b**,**c**) The experiment was repeated for selected doses of ibuprofen (25 µg/mL and 50 µg/mL) (**c**) or adequate amount of ethanol-ibuprofen solvent (**d**) on 6 populations at 4 different time points: 1 day (24 h), 3 days (72 h), 7 days and 10 days. Diagrams present mean optical density (OD) was measured at 570 wave length. (**d**). The effect of ibuprofen on hBM-MSCs proliferation determined with BrdU assay. Bars present mean (±SEM) relative cell proliferation ratio. Samples were analyzed in quadruplicates. The experiment was performed on the cells derived from 6 independent donors. Statistical analysis was performed by the Wilcoxon matched-pairs signed-rank test in the groups of related data with abnormal distribution, and by the Student’s *t*-test in the groups of related data with confirmed normal distribution. *p* < 0.05 was assumed to be statistically significant; ns—statistically not significant. No statistical differences were determined between groups in the MTT experiment (**b**,**c**).

The effect of ibuprofen on hBM-MSCs viability was determined using the MTT test. In preliminary studies, we analyzed the effect of six concentrations of ibuprofen (25 µg/mL, 50 µg/mL, 100 µg/mL, 200 µg/mL, 400 µg/mL, and 800 µg/mL) in five different time points (24 h, 48 h, 72 h, 96 h or 120 h) on three cell populations. Based on this data, hBM-MSCs survival curves were plotted (Figure 2a).

The decrease in the survival of hBM-MSCs was observed with the increase in the concentration of ibuprofen and the duration of the experiment. Detailed analysis showed that ibuprofen reduces the cell viability in a dose-dependent manner. Pearson’s correlation coefficient (r) showed a strong dependence of cell viability on the applied ibuprofen dose. At all tested treatment times, r > 0.89 (mean r = 0.93). However, no such strong relations were observed between cell viability and treatment time (Table 1). Pearson’s correlation coefficient ranged from r = 0.24 (for 1.0 ug/mL ibuprofen concentration) to r = 0.72 (for 2.0 ug/mL ibuprofen concentration) across the ibuprofen concentrations tested, with a mean r = 0.54.

The experiment was repeated for selected doses of ibuprofen (25 µg/mL and 50 µg/mL) on six populations. This time, four different time points were tested: 1 day (24 h), 3 days (72 h), 7 days and 10 days. The obtained results confirmed the previous data (Figure 2c) and the treatment time did not affect hBM-MSCs survival. The maximum decrease in cell viability was observed after 10 days of treatment for 50 µg/mL of ibuprofen (92% relative viability). There were no statistically significant differences in the cell survival rates between the groups tested (CTRL, IBU25, IBU50).

### 3.3. Ibuprofen Does Not Affect the hBM-MSCs Proliferation

To determine the effect of ibuprofen on hBM-MSCs proliferation, a BrdU assay was performed. The study was conducted on six populations treated for 72 h with two different doses of ibuprofen. Analysis of the results revealed no statistically significant correlation between control and treated groups (Figure 2d). In addition, no proportional relation was observed between the concentrations of the tested drug. The data obtained from the studied populations did not show a common trend in proliferation rate after ibuprofen treatment and ranged from 80 to 120% of control values. The mean % value for the IBU25 group was 108% of CTRL, and for IBU50 was 96% of CTRL.

### 3.4. Ibuprofen in Therapeutic Doses Substantially Reduce the Secretion of PGE_2_ by hBM-MSCs

**Figure 3:**
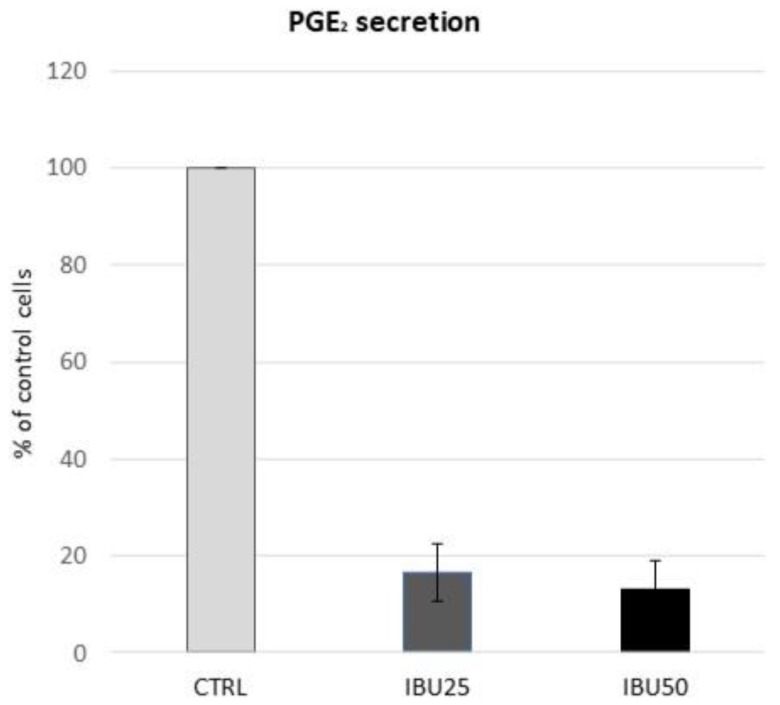
hBM-MSCs secretion of PGE_2_ after ibuprofen treatment. PGE_2_ concentration in hBM-MSCs supernatants collected from 6 different populations was quantified with ELISA immunoassay after 24 h of incubation with 25 µg/mL or 50 µg/mL ibuprofen. Relative PGE_2_ concentration in supernatants from cells treated with ibuprofen versus control (100%).

To confirm that doses of ibuprofen used in this study provide the expected biological effect in terms of cyclooxygenase inhibition, an ELISA assay was performed to measure PGE_2_ production. The analysis revealed that both concentrations of ibuprofen caused inhibition of PGE_2_ secretion in a dose-dependent manner in hBM-MSCs (Table 2). In relation to the control, the mean secretion was decreased by 83.5% in the IBU25 group, and by 87% in the IBU50 group (Figure 3). The obtained inhibitory effect was so potent that in both the IBU25 and IBU50 groups, the OD of some samples was below the detection level.

### 3.5. Ibuprofen Does Not Affect the hBM-MSCs Migration

**Figure 4:**
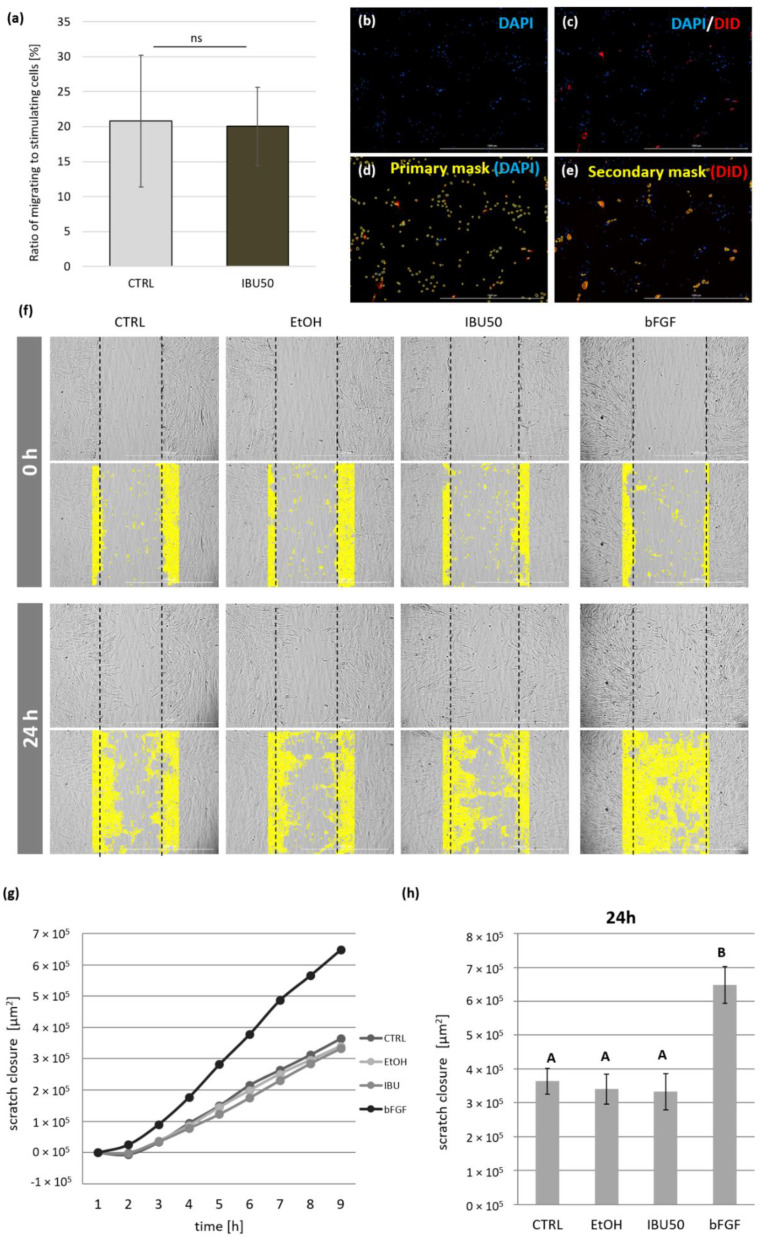
The effect of ibuprofen (50 µg/mL) on the migration of human BM-MSCs. (**a**) Transwell migration assay. The experiment was performed in duplicates on 6 hBM-MSCs populations. The results are presented as mean (± SEM) percentage of migrated (DID+/DAPI+) cells per number of stimulating cells (DID−/DAPI+). Data analyzed with the Wilcoxon matched-pairs signed-rank test. *p* < 0.05 assumed as statistically significant, ns—statistically not significant. (**b**–**e**) View of the representative well after 72 h of migration. All images represent the same field of view. (**a**) Cell nuclei stained with DAPI (blue color, both stimulating and migrating cells); (**b**) migrating cells stained with DID (red color); (**c**) the view with primary mask (cell nuclei) marked in yellow by software; (**d**) the view of secondary mask (DID) in yellow by software. (**f**–**h**) The scratch assay. (**f**) Images representing the behavior of mesenchymal stromal cells during a scratch test and the way of analysis. Each column presents pictures of the same field of view at 0 time point (upper two rows) and at 24 h time point (lower two rows). Analysis was performed within a plug marked by the software (yellow color). The dashed lines indicate the “scratch” margins at point 0 for each field of view. (**g**) The mean “wound” closure was documented every 3 h for 24 h. Scratch covering is presented in µm^2^ in time (/h) in relation to the time point 0. CTRL-Opti-MEM™ medium was supplemented with 4% of FBS, EtOH (solvent control), IBU50 (addition of ibuprofen in 50 µg/mL), and bFGF (positive control, 20 ng/mL). (**h**) Bars represent mean values (±SEM) of scratch covering (µm^2^) at 24 h time point. Data analyzed using ANOVA with Tukey post-hoc test (normal data distribution confirmed using Shapiro–Wilk test). Bars marked with different uppercase letters indicate that the groups differ significantly, *p* < 0.001. Bars marked with the same letters do not differ significantly with each other (*p* > 0.05). Data collected during 4 experimental sets, MSCs from 5 different donors used, total n = 9. Test performed in quadruplicates.

We first examined how ibuprofen affects MSCs in terms of their ability to stimulate targeted migration. In this experiment, MSCs migrated through cell culture inserts (8 µm pore size) towards untreated or ibuprofen-treated MSCs. There were no statistically significant differences in the directed migration level between the study group (IBU50) and the control (CTRL) (Figure 4a,b). The individual results obtained from studied populations did not show a common trend after ibuprofen treatment. The mean level of migration for the group treated with ibuprofen was 116.7% of the control (100%).

Next, to assess the effect of ibuprofen on the overall mobility of MSCs, a scratch assay was performed, in which the “wound” closure was documented every 3 h for 24 h, and statistical analysis was performed at the 24 h time point. The automated microscope and software used allowed for successful evaluation of the process of migration (Figure 4c). The results indicate that neither the solvent (ethanol) nor ibuprofen in a dose of 50 µg/mL did not affect the mobility of BM-MSCs during the course of experiment (*p* > 0.05 at 24 h). The addition of bFGF (positive control) significantly increased the coverage of the scratch area in all cases (*p* < 0.001 in comparison with all groups using ANOVA with the post-hoc Tukey test (Figure 4d,e).

### 3.6. Ibuprofen Affects hBM-MSCs Secretion Profile

The secretion of trophic factors by the MSCs is the basis of their immunomodulatory properties and regenerative potential. Therefore, in this study, changes in the BM-MSCs secretome after ibuprofen treatment were evaluated using two different experimental methods. The concentration of different proteins was measured in culture medium collected after the last 24 h of treatment with 25 µg/mL and/ or 50 µg/mL ibuprofen (the whole duration of treatment lasted 72 h).

#### 3.6.1. Ibuprofen Changes Protein Secretion in BM-MSC

**Figure 5:**
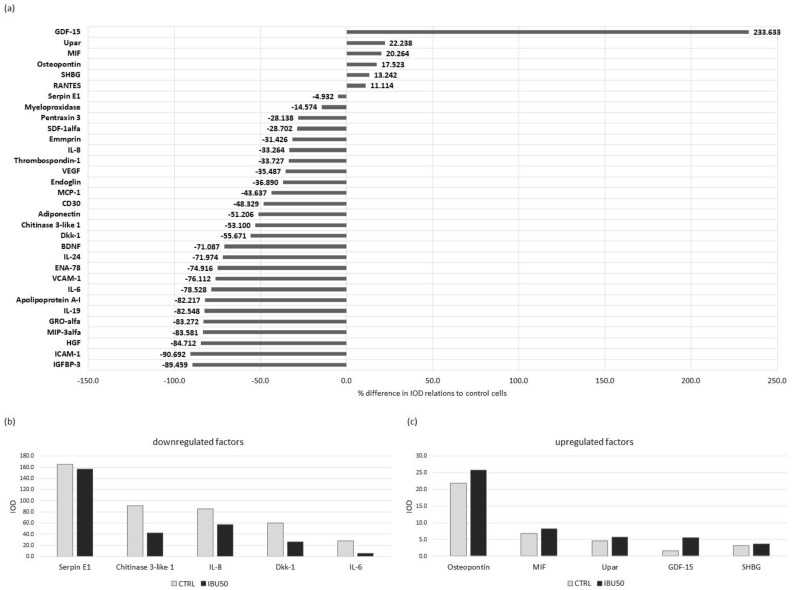
Proteome Profiler analysis of cytokines and chemokines secreted by hBM-MSCs. Semi-quantitative evaluation of secretion levels of over 100 human cytokines produced by BM-MSCs cultured in control medium (CTRL) or in medium with 50 µg/mL of ibuprofen for 72 h. For this analysis, supernatants collected from 6 BM-MSCs donors were pooled. (**a**) Cytokines levels changed after ibuprofen treatment in comparison to the control group. Data are presented as a percentage of control MSCs secretion (100%). (**b**) Factors decreased after ibuprofen treatment which was most abundantly secreted by untreated cells (factors which gave signal of highest density). (**c**) Factors which increased after ibuprofen treatment. Bars represent IOD (integrated optical density) of antibodies–dots measured by chemiluminescent detection.

First, we performed semi-quantitative Proteome Profiler screening of over 100 proteins in the secretome of BM-MSCs treated with 50 µg/mL of ibuprofen (pooled media collected from cultures of six populations) relative to the secretome of control cells. The assay revealed changes in the levels of multiple secreted factors including HGF, IL-6, IL-8, VEGF, MCP-1, and others (Figure 5a).

Interestingly, the secretion of most of the examined factors was decreased, with the strongest inhibition of secretion observed for ICAM-1 (90.6% decrease from control), IGFBP-3 (89.4% decrease), and HGF (84.7% decrease). There were only six factors with increased secretion: GDF-15 (263.3% increase compared to control), uPAR (20.2% increase), macrophage migration inhibitory factor (MIF) (20.2% increase), osteopontin (17.5% increase), sex hormone binding globulin (SHBG) (13.2% increase) and CCL5 (RANTES) (11.1% increase). The most highly secreted factors were serpin E1 (IOD 165.1 in control and 157.04 in IBU50), chitinase 3- like- 1 (IOD 90.5 in control and 42.4 in IBU50), CXCL8 (IL-8) (IOD 85.2 in control and 56.8 in IBU50), Dkk-1 (IOD 59.9 in control and 26.5 in IBU50), and IL-6 (IOD 27.5 in control and 5.9 in IBU50) (Figure 5b). The highest concentrations of increased secretory factors were obtained for osteopontin (IOD 21.8 in control and 25.6 in IBU50).

#### 3.6.2. Ibuprofen Significantly Reduces the Secretion of Selected Cytokines

**Figure 6:**
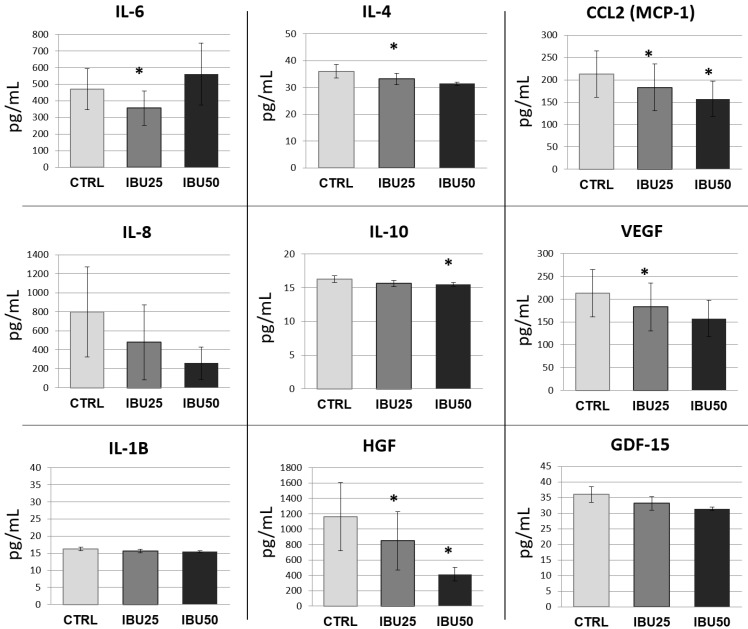
Analysis of proteins secretion. Luminex quantitative evaluation of IL-1β, IL-4, IL-6, IL-8, IL-10, CCL-2, GDF-15, HGF, and VEGF secretion levels (pg/mL) produced by BM-MSCs cultured in control medium (CTRL) or in medium with 25 µg/mL or 50 µg/mL of ibuprofen. The experiment was performed in duplicates, and on cell populations from 6 donors. Statistical analysis was performed by the Wilcoxon matched-pairs signed-rank test and by the Student’s *t*-test (depending on data distribution determined by the Shapiro–Wilk test). * *p* < 0.05.

To quantitatively analyze the concentration of selected immunomodulatory factors in the collected hBM-MSCs culture media, Luminex immunoassay was performed. Proteins for analysis were selected based on Proteome Profiler results and literature data, indicating their participation in MSCs immunomodulation [43]. The concentrations of the following proteins were determined: IL-1β, IL-4, IL-6, IL-8, IL10, GDF-15, HGF, MCP-1, and VEGF. Similar to the Proteome Profiler results, we observed a decrease in secretion of most of the factors examined after treatment with ibuprofen (Figure 6).

The decrease in secretion was dose-dependent for all tested factors, except for IL-6, where an increase in secretion was observed in the IBU50 group (mean 561.6 pg/mL) compared to the control (mean 469.2 pg/mL) and IBU25 (mean 356.5 pg/mL) groups. For MCP-1 and HGF, the decrease in secretion was significant for both ibuprofen doses tested (for MCP-1, the decrease from 213.05 pg/mL in control to 183.03 pg/mL in IBU25 and 157.5 pg/mL in IBU50 groups, and for HGF, the decrease from 1162.7 pg/mL in control to 849.04 pg/mL in IBU25 and 412.5 pg/mL in IBU50 groups). For IL-4 and VEGF factors, a statistically significant decrease in secretion compared to control was observed only in the IBU25 group (a decrease from 36.0 pg/mL in control to 33.1 pg/mL in IBU25 for IL-4, and from 213.05 pg/mL in control to 183.03 pg/mL in IBU25 for VEGF, respectively). For IL 10, statistical significance was found only in the IBU50 group (a decrease from 16.25 pg/mL in CLRL to 15.5 pg/mL in IBU50). For the factors, IL-1β, IL-8 and GDF-15, the differences between the groups were not statistically significant. The total protein concentration in collected cell culture media did not differ statistically between studied groups.

## 4. Discussion

Non-steroidal anti-inflammatory drugs (NSAIDs), due to their anti-inflammatory, analgesic and antipyretic properties, are one of the most commonly used medications worldwide [44]. Currently, the most popular drug among this group is ibuprofen, widely used mainly in the symptomatic treatment of mild to moderate pain of various origins [45]. The appearance of pain is a frequent symptom of ongoing inflammation resulting from physical or biological factors. Inflammation development is associated with activation of repair mechanisms in the form of a multi-step cascade of events aimed at protecting the body from infection, reconstructing damaged structures and restoring homeostasis. A certain role in this process is played by MSCs, which are not only able to differentiate into various types of cells that build the human body, but above all, have immunomodulatory capabilities [46]. NSAIDs have been proven to interfere with the natural process of tissue regeneration and their adaptation to stress factors [47,48,49]. The action of ibuprofen, like other NSAIDs, relies on the inhibition of synthesis of prostaglandins, including PGE_2_. These molecules are crucial in the development of inflammatory reactions. NSAIDs act through inhibiting two cyclooxygenase isoforms: COX-1 and COX-2. It has been shown that the ability of MSCs to inhibit T cells and interact with macrophages requires the presence of PGE_2_ [50]. Therefore, inhibition of this prostaglandin production may result in a reduction of MSCs therapeutic potential. However, to date, there are no data on the effect of NSAIDs on the ability of MSCs to shape the immune response. Therefore, the main research objective of the present study was to evaluate the in vitro effects of therapeutic doses of ibuprofen on the properties of human bone marrow derived MSCs.

One of the core features of MSCs that allows their therapeutic use is their ability to proliferate while maintaining in an undifferentiated state. It was postulated in the literature that PGE_2_ may be involved in the regulation and maintenance of these functions. Lee et al. (2016) [20] showed that the inhibition of PGE_2_ production with the use of COX-2 or mPGES-1 inhibitors (an enzyme for PGE_2_ synthesis) resulted in a reduction of proliferation of hMSCs from adipose tissue and umbilical cord blood (UCB). Additionally, it was shown that PGE_2_ stimulated the proliferation of UCB-MSCs [51]. More detailed analysis by Kleiveland et al. has shown that PGE_2_, depending on the concentration, may have an opposing effect on MSCs proliferation [52]. We have demonstrated that the inhibition of PGE_2_ production with ibuprofen at a dose of 25 or 50 µg/mL did not cause changes in the proliferation rate hBM-MSCs in relation to the untreated cells which seem to be in contrast with some of the previously mentioned studies. These discrepancies may result from the COX-inhibitors concentrations used in different studies, and thus the level of PGE_2_ secretion achieved. Ibuprofen concentrations chosen in our study did not differ from the maximum serum concentrations during standard pharmacotherapy [39]. Meanwhile, doses of celecoxib used in the study of Lee et al. (2016) were at least several times higher than therapeutic plasma concentrations [20]. This hypothesis was confirmed by the Müller et al. (2011) [32], who concluded that the effects of NSAIDs on MSCs mainly depend on the concentrations used. In their article, NSAIDs at lower concentrations (0.1–1 μM for celecoxib and meloxicam and 10–50 μM for flunixin) exert a positive effect on equine MSCs proliferation [32]. In addition, in our study, we demonstrated that in the preliminary experiments the higher doses of ibuprofen negatively affect MSCs viability in MTT assay. This test is sometimes used to evaluate cell proliferation. Therefore, we can assume that larger doses of ibuprofen inhibit MSCs proliferation. However, in our opinion, in vitro studies should use drug doses which correspond to the clinical situation. If not, their potential for translation (to clinical conditions) is substantially limited.

Another important phenomenon that determines the proper course of inflammatory and healing processes is cell migration. Cells of the immune system and progenitor cells involved in regenerative processes have the ability of directed migration to an inflamed site. Previous studies have shown that MSCs are able to migrate to the site of injury, but also provide chemotactic signals to other cells. In our study, we examined whether ibuprofen affects these abilities. First, we evaluated the overall mobility of MSCs using a scratch test. We demonstrated that the addition of ibuprofen at the doses found in the patients’ serum did not impair MSCs migration. Previously, Lu et al. (2017) showed that PGE_2_ increased MSCs’ migration in a scratch test [21], which seems to be in contrast to our results. However, our data together with those presented by Lu at al. indicate that PGE_2_ may be one of the potential stimulators of MSCs migratory capacity, but in case of reduced PGE_2_ concentration, the presence of other factors is sufficient to maintain the basal mobility of this population. The next issue to consider is directed migration and chemotaxis. It is known that MSCs secrete chemotactic factors to which they are able to respond. We confirmed this in our previous work [53], where we showed that MSCs, regardless of the source and donor age, very strongly stimulate the directed migration of BM-MSCs in the transwell assay. The migration protocol we proposed in the present study allowed us to assess the impact of ibuprofen on hBM-MSCs secretion in terms of stimulating the directed migration. The assay was designed to study the recruitment of circulating, or local endogenous mesenchymal cells. We have demonstrated that MSCs pretreated with IFN-γ and then treated with ibuprofen stimulate the directed migration of BM-MSCs to the same extent as control cells (stimulated with IFN-γ but not treated with ibuprofen).

To investigate the impact of ibuprofen on secretory activity of MSCs, we performed semi-quantitative and quantitative analyses of the MSCs secretome. MSCs are known to secrete numerous bioactive factors and this activity is considered crucial for their immunomodulatory and pro-regenerative abilities. First, we evaluated the effect of ibuprofen (50 µg/mL) on the secretion of a wide range of cytokines using the Proteome Profiler XL Cytokine Array. The results indicated that the inhibition of secretion was the dominant direction of change. Among the factors whose secretion changed by at least 20% with ibuprofen treatment, secretion of 24 decreased, while it increased in only three of them.

Among the factors where the concentration in MSCs culture media increased after ibuprofen treatment were: osteopontin (OPN), macrophage migration inhibitory factor (MIF) and growth/differentiation factor 15 (GDF-15). The first of these, OPN, is abundantly secreted by MSCs. Under physiological conditions, OPN synthesis decreases in an inflammatory environment, mediated by PGE_2_ secreted by MSCs. This indicates the OPN as a signal modulated by MSCs according to their activation status [54]. The increase in OPN secretion observed in our study after treatment with a drug inhibiting PGE_2_ production seems to be consistent with these literature data. The protein where secretion increased most after ibuprofen treatment in the Proteome Profiler analysis was the inflammatory biomarker, GDF-15. However, these data were not confirmed by quantitative analysis, where no statistically significant difference between groups was observed. The reason for this discrepancy in the obtained results may be because of a relatively low level of secretion of this protein by MSCs.

Among the factors where the concentration in supernatants decreased after ibuprofen treatment, a number of factors are associated with immunomodulation and regeneration processes i.e., IL-6, IL-8, CCL2, HGF and VEGF. These were the molecules of particular interest in our study; therefore the results obtained were verified by the more accurate, quantitative Luminex method. For all the factors mentioned above, except of IL-8, it was confirmed that the secretion was significantly reduced by at least one tested dose of ibuprofen compared to control. The secretion of IL-6 was decreased in the ibuprofen group (in comparison to the untreated group) only when a lower dose was used. IL-6 is one of the most abundantly secreted factors by MSCs [40], which was confirmed in our results. IL-6 is a key mediator involved in the maturation and differentiation of immune system cells such as macrophages, lymphocytes and dendritic cells. Although IL-6 is considered a pleiotropic factor, its activity is mainly described as pro-inflammatory. It plays a role both in acute inflammation e.g., in the cytokine storm in COVID-19 and in chronic conditions e.g., in numerous autoimmune diseases [55].

The next factor decreased by ibuprofen treatment was CCL2. This is a chemokine that is responsible for recruiting monocytes to the site of injury. Reduced CCL2 secretion may result in impaired recruitment of monocytes, which may have further consequences. On the one hand, it may reduce the severity of the inflammatory process, but on the other hand, it may impede tissue healing, as monocyte-derived macrophages play an important role in this process. It was demonstrated that CCL2-overexpressing MSCs display higher recovery potential after transplantation to mice with induced acute stroke than unmodified MSCs [56]. HGF and VEGF are also factors involved in the healing and regeneration processes [57]. HGF promotes proliferation and motility of many cell types and suppresses chronic inflammation and fibrosis. VEGF is a well-known pro-angiogenic factor required for the proper course of tissue recovery after injury. Therefore, our data indicate that ibuprofen, in therapeutic doses, induces significant changes in the secretory profile of MSCs, which may contribute to the attenuated pro-regenerative effect elicited by this population. These data are in agreement with previous results from preclinical and clinical studies indicating that ibuprofen can impede healing processes [26,27,28,29,30,31]. Moreover, Galleli et al. [58] demonstrated that ibuprofen treatment induced a statistically significant decrease in the IL-6 and VEGF concentration and did not modify IL-8 concentration in synovial fluid in patients with knee osteoarthritis, which is very consistent with our data.

In conclusion, our results indicate that ibuprofen at concentrations that are achieved in the serum of patients during standard pharmacotherapy does not significantly affect the proliferative and migratory capacity of human bone marrow-derived MSCs. However, we demonstrated that ibuprofen in these doses does have an impact on the MSCs secretion profile. The observed changes indicate that ibuprofen reduces the secretion of factors related to regenerative processes (like HGF, VEGF, CCL2). These results suggest that the use of ibuprofen after MSCs transplantation might have an impact on the outcome of the procedure. Possibly, the inhibitory effect of ibuprofen on the secretion of regenerative factors by MSCs is partly responsible for the previously described impairment of the healing processes by NSAIDs. However, verification of these hypotheses requires further studies.

## Figures and Tables

**Table 1:** Pearson’s correlation analysis was performed to check whether there was a linear relation between cell viability and the ibuprofen dose or treatment time. The effect of ibuprofen on hBM-MSCs viability was evaluated with MTT assay. Cells from 6 donors were treated with different concentrations of ibuprofen (25 µg/mL, 50 µg/mL, 100 µg/mL, 200 µg/mL, 400 µg/mL, and 800 µg/mL) for, respectively, 24 h, 48 h, 72 h, 96 h or 120 h. The experiment was performed in triplicates.

Time Dependent Correlations	
Ibuprofen Concentration [ug/mL]	Pearson’s Correlation Coefficient (r)
25	0.46
50	0.5
100	0.24
200	0.72
400	0.65
600	0.7
800	0.53
average	0.54
Dose dependent correlations	
treatment time [h]	Pearson’s correlation coefficient (r)
24	0.9
48	0.96
72	0.95
96	0.94
120	0.89
average	0.92

**Table 2:** hBM-MSCs secretion of PGE_2_ after ibuprofen treatment. PGE_2_ concentration in hBM-MSCs supernatants collected from 6 different populations was quantified with ELISA immunoassay after 24 h of incubation with 25 µg/mL or 50 µg/mL ibuprofen. The amount of PGE_2_ (pg/mL) secreted by each study population (assay range: 7.8–1000 pg/mL).

PGE2 Concentration [pg/mL]			
	CTRL	IBU25	IBU50
Population 1	12.59	underdetection	underdetection
Population 2	525.54	12.48	11.62
Population 3	68.21	8.34	9.92
Population 4	38.55	8.84	8.58
Population 5	32.14	9.15	underdetection
Population 6	28.78	underdetection	underdetection
mean	117.63	9.71	10.04
SD	200.66	1.88	1.52
SEM	81.92	0.77	0.62

## Data Availability

Data is contained within the article.
